# Notes

**Published:** 1900-07

**Authors:** 


### NOTES.

In October of every year upward of 3,000,000 geese are driven to market at Warsaw, Poland, to be sold, and most of them are exported to Germany. The geese often come from remote provinces, and have to travel long distances over rough roads. In order to protect their feet they are "shod" by a peculiar process. First, they are driven through tar poured upon the ground, and then through sand. After the operation has been repeated several times the feet of the geese become covered with a hard crust which effectively protects them.

It is said that nearly one hundred and fifty veterinarians in various parts of the country presented themselves for examination before the army examining board for positions as army veterinarians.

A Chicago architect has made plans for a mansion for stray dogs, and if the designs shall be approved the edifice will be erected at once.

Moral rhymes—

I. THE GOAT.

The goat is not regarded as
A noble beast, and it
Has never won distinction for
An undue share of wit.

Compare the homely goat with man;
How godlike does he stand;
How pitiful the beast becomes
And how absurdly planned.

Yet, while we look upon the goat
As neither fair nor wise,
It does not live by taking pills
Instead of exercise.

II. THE MULE.

Man is the greatest work of God,
The oxen are his slaves;
He lures the lightning from the cloud
And harnesses the waves.

To him alone a soul is given,
And he receives a birth;
The glorious hope of joy in heaven
When he is done with earth.

Man is sublime; the homely mule
Is made of poorer stuff;
But mules quit drinking, as a rule,
When they have had enough.

—S. E. KISER, in *Chicago Times-Herald* (*Rider and Driver*, June 2, 1900).

Veterinary obstetrics should gain apace in keeping with the many valuable contributions to this all-important part of our work in the profession. Dalrymple's contribution, of much worth, will be now added to by a translation of Fröhner and Bayer, which, added to that of Fleming's, which covers the great work of St. Cyr, makes English literature rich in this direction.

Epizootic cellulitis is raging in Wisconsin to such an extent as to seriously interfere with business where horses are used.

Calves may be prevented from sucking cows, and cows may be broken of the habit of sucking themselves, by washing the udders of the cows with a decoction of cayenne pepper. A tablespoonful of cayenne pepper steeped in a pint of water will make a liquid strong enough to disgust the sucking calf or the self-sucking cow. Apply the decoction to the teats and the udder with a rag. Generally but one application is needed. Persistent suckers will rarely tackle the pungent substance a second time.—*Correspondence New York Farmer* (*Jersey Bulletin*, June 27, 1900).

At the Munich meeting of the Tuberculosis Commission, held under the auspices of the German Naturforacher und Aerzte-Versamiung, Italo-Tonta, of Milan, summarized in an admirable way the regulations which should be established by the authorities for the prevention of tuberculosis. These regulations are so concisely and clearly stated that they might well be embodied in a tract and scattered broadcast throughout the world. They are as follows :

1. The periodic disinfection of all localities much frequented by the public, especially rooms in which many individuals congregate, such as schools, society-rooms, churches, cafés, restaurants, hotels, orphanages, barracks, libraries, convents, hospitals, dispensaries, stores, tramway and railway cars, and cabs.

2. The prohibition of spitting on the floors in rooms and public conveyances; the placing of cuspidors in parks and other public places, and in vehicles of transportation.

3. The establishment of special playgrounds for children, in order to avoid their playing in localities which phthisical patients might visit.

4. The disinfection and whitewashing of rooms where a case of phthisis or a death from that disease has occurred.

5. The annual medical inspection of persons frequenting schools, academies, offices, factories, etc. Any cases found should be reported to the authorities.

6. The establishment of people's sanitariums.

7. The hygienic instruction of the tuberculous, so that they may be able to protect themselves and those coming in contact with them.

8. The isolation of the phthisical cases in military and general hospitals—if possible, the erection of separate pavilions.

9. The prohibition of the bathing of the tuberculous with healthy persons; the establishment of separate bath-houses for the former, under medical supervision.

10. The removal of all tuberculous individuals from the schools and their transfer to colonies in the country, where they may be treated.

11. The formation of committees with the object of sending the children of poor persons that are suffering with tuberculosis, or that have died of that disease, to the country, in order to remove them from the infected houses. The children of rich families should also be removed from their homes for a certain length of time.

12. The improvement of the hygienic and dietetic conditions of the poorer classes, by the erection of public kitchens, wayfarers' lodges, bath-houses, etc.

13. Philanthropists should make it their object to improve the nutrition and hygiene of individuals in poor families in which tuberculosis has occurred.

13*a*. The linen of tuberculous persons must be disinfected before being brought into contact with the linen of others.

14. The marriage of very young persons whose appearance suggests that they are inclined to tuberculosis should be opposed. Persons in whose sputum bacilli are present should be prohibited from marrying.

15. The compulsory periodic examination of domestic animals which might become tuberculous.

16. The monthly inspection of stables; supervision of the hygiene of the kitchen, of milking and milking vessels; scrupulous care in creameries.

17. The supervision of markets and abattoirs.

17*a*. The erection of stations at the borders of countries for the inspection of imported animals.

18. Strict regulations regarding products of factories.

19. The giving of weekly lessons in hygiene at all public schools.

20. Each child at school must have its own drinking cup and its own towel. School children should not kiss each other.

21. Instructions to second-hand dealers in books, clothing, etc., to have their wares disinfected. Disinfection of library books, as well as of objects that serve for school or general use, must also be performed at certain intervals.

It may not be possible to carry out all the suggestions contained herein, but they form a very excellent guide for modern sanitary efforts.—*The Medical Record.*

Dogs in Hamburg are taxed according to size—the bigger the dog the higher the tax.

It is evident on looking over the reports of State Veterinary Examining Boards that several colleges of North America did not give sufficient education in the elementary subjects of chemistry, physiology, zoötechnics, sanitary medicine, and meat- and milk-inspection.

It is remarkable to what extent the ox, when slaughtered, is utilized. Not so very long ago fully 40 per cent. of the carcass was wasted. It may be said that to-day nothing is wasted; everything, from the horns to the tail, is turned into money. The blood is used in the refining of sugar, or is hardened and employed in the manufacture of door-knobs and handles; the skin goes to the tanner; the horns and hoofs are turned into combs and buttons, the shin-bones into backs of clothes brushes. The bones of the forefeet are worth $25 a ton, being made into collar buttons, umbrella handles and various novelties after the marrow has been boiled out of them. The small bones are burned instead of coal. From each foot a considerable quantity of oil is extracted; the tail is made into soup. The hair goes to the mattress-maker and upholsterer; the fat to the oleo makers; the intestines are used as sausage wrappers or are sold to gold-beaters. Even the undigested stuff in the stomach is turned to account, being made into paper. If anything is left over it is turned into glue or is put on land as a fertilizer.

The establishment of an Irish veterinary college at Dublin under similar auspices as exist in those at London, Glasgow, and Edinburgh, under royal direction, does not awaken a deep sense of interest in Dublin among local veterinarians, owing to the establishment of a subscriber's membership, entitling the latter at a small annual fee to almost unlimited veterinary services. The abuse of free clinics at many of our American veterinary colleges works an injustice to local members of the profession, and should be abolished.

The Lacey Act of the last Congress, regulating the protection and importation of birds and animals not otherwise provided for, will be placed under the direction of Dr. T. S. Palmer. The further importation of the English house sparrow, the starling, the fruit bat or flying fox, and mongoose, known also as the ichneumon or Pharaoh's rat, *is absolutely prohibited, and permits for their importations will not be issued under any circumstances.*

Dr. W. E. A. Wyman, of Burlington, Wis., has translated the obstetrical part of the recent work of Fröhner, of Berlin, and Bayer, of Vienna. It will be published by the well-known publishing house of W. R. Jenkins, New York.

